# The role of individual differences and attitude in willingness to participate in TMS studies

**DOI:** 10.3758/s13428-025-02623-4

**Published:** 2025-03-10

**Authors:** C. Lolansen, C. J. Howard, S. Mitra, S. P. Badham

**Affiliations:** 1 School of Psychology, University of Nottingham, University Park Campus, East Dr, Nottingham, NG7 2RD UK; 2 Department of Psychology, Nottingham Trent University, Nottingham, NG1 4BU UK

**Keywords:** Transcranial magnetic stimulation/TMS, Ageing, Eligibility, Barriers to Participation, Participant Recruitment

## Abstract

Finding neurophysiological research participants can be challenging, especially when the technology used in the research study is less known, such as transcranial magnetic stimulation (TMS). Despite this well-known phenomenon, there is limited literature investigating the factors involved in willingness to participate and perceived barriers from the potential participants’ perspective. This paper explored the relationship between individual differences, attitudes toward TMS, and willingness to participate in TMS research alongside perceived barriers to participation and concerns when considering participating. The findings suggest that participants who had more positive attitudes towards TMS were more willing to participate. Participants frequently reported being concerned about safety, including risks and side effects. For barriers in terms of safety parameters, the number of participants who were eligible based on their TMS safety screening questionnaire was low, particularly for older adults. These findings are discussed in the context of the literature, and practical guidelines are provided for researchers looking to plan TMS recruitment.

**Supplementary Information:**

The online version contains supplementary material available at 10.3758/s13428-025-02623-4.

## Introduction

Advancements in psychology and scientific methods have paved the way for studying human behaviour using neuroscientific methods with a focus on the relationship between the brain and behaviour. A variety of neuroscientific methods have been used to investigate the complex relationship between these two components, one of which is transcranial magnetic stimulation (TMS). TMS is a non-invasive brain stimulation method that applies transient magnetic fields which affect the electrical activity of neuron assemblies. The effects of TMS are induced by discharging an electrical current through a wire coil. This produces a transient magnetic field around the coil, which depolarises neurons and alters cortical excitability (Wagner et al., 2007). This can alter brain activity in the affected areas in an inhibitory or facilitatory manner (Nollet et al., 2003). TMS offers researchers a means of testing causal theories of brain–behaviour links. Some of this research has clinical implications, with evidence suggesting that TMS can be a viable treatment intervention for a variety of clinical conditions, such as dementia (Cantone et al., 2014), Alzheimer’s disease (Nardone et al., 2014; Dong et al., 2018), Parkinson’s disease (Nardone et al., 2020), schizophrenia (Cole et al., 2015), post-traumatic stress disorder (Yan et al., 2017), depression (Cotovio et al., 2023; Perera et al., 2016), and anxiety (Cirillo et al., 2019).

However, while TMS is generally well tolerated by most participants, it is important to note the potential risks and side effects of TMS (Taylor et al., 2018). These range from minor side effects such as those caused by the stimulation (headaches or localised pain/discomfort following stimulation) or by the manner in which the participant receives the stimulation (discomfort or pain due to postural rigidity during the stimulation). As reported by Taylor et al. (2018), less than 2% of participants in clinical trials discontinue participation due to pain-related side effects. While there is a risk of severe side effects, such as seizures or hearing impairment, the risk of these is very low when appropriate safety measures are followed. Stringent safety guidelines offer clear parameters for researchers to mitigate the risks of TMS, such as appropriate setups for participant safety and comfort, hearing protection, and only administering stimulation within accepted safety parameters (Rossi et al., 2021). Safety screenings are conducted prior to administering TMS to assess potential risk factors in individual participants, commonly relating to seizure risk (such as history of epilepsy, a range of neuropsychiatric conditions, alcohol or other substance use, sleep deprivation, some medications, and medical conditions known to lower seizure thresholds) and general risks associated with procedures involving magnetism (such as metal or shrapnel in the body, medical infusion devices, or metal implants, Rossi et al., 2009; 2021). When these safety guidelines are adhered to, the risk of severe side effects is very low.

The breadth of applications for research and clinical purposes that TMS offers is one of its most notable strengths. However, as outlined above, TMS is a method for non-invasive brain stimulation. It is subject to stringent safety requirements and may not be suitable for all individuals (Rossi et al., 2021). This presents one notable limitation when it comes to recruiting for research studies. Much of psychology research is reliant on volunteer participants from the public or student populations. Acquiring a sufficient number of participants is important for quantitative research studies to ensure the statistical analyses are appropriately powered (Baker et al., 2021). Recruiting enough participants can be challenging, and recruitment success can vary greatly due to target population, resources, or logistics. TMS research, while non-invasive, also has persistent recruitment challenges. To understand the recruitment difficulties for TMS research, it is crucial to understand potential participants’ deliberation processes when deciding whether to participate in a research study.

Research demonstrates that recruitment challenges can be particularly pronounced for medical studies (McDonald et al., 2006). Some research has attempted to explore these recruitment challenges from the perspective of participants and has provided valuable insight into people’s decision-making processes when considering participating in a research study. Several factors can influence an individual’s willingness to participate in a research study, such as understanding the research and its potential benefits (Dunlop et al., 2011) and perceived relevance of the research to the individuals themselves (Glass et al., 2015). As TMS is a lesser-known research method, potential participants may be particularly wary of participating in a TMS research study due to their lack of understanding of the method. This may be the case for TMS even when compared to other methods such as fMRI or EEG, as TMS alters brain activity which can be concerning or off-putting for potential participants. Concerns about risks and side effects of participating in research studies are common barriers to participation in other literature (Dunlop et al., 2011; Halpern et al., 2003). Similarly, how “safe” a study is perceived to be has also been linked to willingness to participate (Chu et al., 2015). One could extrapolate that understanding of, or just familiarity with, the method used in the research study and fear may be linked, and that these two in turn, affect willingness to participate. However, these factors have not been investigated specifically in the context of TMS. Understanding these potential links could provide valuable insight into recruitment for TMS researchers and other scientists using methods which members of the public may not be familiar with and therefore have concerns about.

One avenue for improving potential participants’ understanding of a research study’s method can be by providing additional information about the study. This may be of particular interest to TMS studies, as there is evidence to suggest that providing additional information can improve willingness to participate (Glass et al., 2015; Kim et al., 2011). Additionally, how information about a participation opportunity is presented may also influence willingness to participate (Latimer et al., 2008). While framing itself has not been extensively studied in the context of research studies, these findings do suggest that there may be an untapped resource in recruitment for research studies in terms of the framing and presentation of research studies to potential participants.

There is also evidence suggesting that individual differences are linked to willingness to participate and general volunteering behaviour, such as demographic factors (Glass et al., 2015), personality (Ackermann, 2019) and health factors (Sedlar & Grezo, 2022). In terms of barriers to participation, potential participants have reported (in)convenience of participating in research studies as a reason for not participating (Halpern et al., 2003). The literature suggests that both perceived risks and willingness to participate in research studies may vary between demographic groups (Svensson et al., 2012). In terms of willingness to participate, while one study found that women are more likely to report being willing to participate in research studies than men (Glass et al., 2015), other research suggests that women are less willing to participate than men (Ding et al., 2007; Otufowora et al., 2021). On the other hand, a study by Trauth et al. (2000) found no significant gender differences in willingness to participate. This suggests that while there may be gender-related factors involved in willingness to participate, the evidence is inconclusive and merits further investigation. In addition to gender, age, and health may also be factors which influence the likelihood of participating. One study found that healthy respondents and young respondents (under the age of 40) were notably more likely to be willing to participate in health research studies (Bouida et al., 2016). However, another study found that older respondents were more likely to report being willing to participate in research studies (Glass et al., 2015), which could potentially be explained by older adults being more concerned about the age-related decline of health and abilities, such as cognitive function (McAlister & Schmitter-Edgecombe, 2016). These contrasting findings demonstrate the need for more research. It is important to note that older adults can be particularly challenging to recruit for TMS research, even if they are willing to participate, due to the safety requirements of participation, such as medicines and medical history (Rossi et al., 2021).

Another factor that may be related to willingness to participate is personality. Evidence suggests that among the Big Five personality traits (see John et al., 1991), extraversion and openness are related to general volunteering behaviour (Ackermann, 2019; Marcus & Schütz, 2005). Furthermore, those who score high on conscientiousness, extraversion, and agreeableness and lower on neuroticism are more likely to volunteer for research studies (Lönnqvist et al., 2007). Other research, however, suggests no link between the Big Five personality traits and willingness to participate in health research (Sedlar & Grezo, 2022). In addition to the Big Five personality traits, individuals who demonstrate greater sensation-seeking are reportedly more likely to participate in activities such as sports (Rowland et al., 1986) and gambling (McDaniel & Zuckerman, 2003) and tend to view activities that are considered risky by people who score lower in sensation-seeking as less risky or dangerous and are more likely to engage in them (Roberti, 2004). Furthermore, individuals who score higher in sensation-seeking are more likely to want to participate in research studies (Oswald et al., 2013), particularly ones deemed “exciting” than those who score lower on sensation-seeking (Thomas, 1989). Table 1 summarises the key findings of the relevant papers.

**Table 1:** List of previous papers investigating factors linked to willingness to participate

Publication	Aspect of willingness to participate	Method	Results relevant for current study
Ackermann (2019)	Personality and volunteering behaviour.	Questionnaire assessing different types of volunteering (formal, informal, and online) and short form of Big Five Inventory (BFI-S).	Greater extraversion predicted volunteering of all three types. Emotional stability predicted formal and online volunteering. Agreeableness predicted more informal volunteering and less online volunteering. Openness to experience predicted less formal volunteering and more online volunteering. Conscientiousness predicted less online volunteering.
Bouida et al. (2016)	Demographic factors, attitude, and willingness to participate in clinical trials.	Face-to-face questionnaire and open-ended questions.	Healthy, young (< 40) participants were more willing to participate. Perceived risk was the primary reason for not wanting to participate.
Ding et al. (2007)	Sex differences in willingness to participate, perceived risk, and distrust of medical researchers.	Double-blind randomised study using hypothetical research study scenarios of various types, durations, and incentives.	Men were more willing to participate than women. Men perceived lower risk of harm than women.
Glass et al (2015)	Demographic differences in willingness to participate.	Telephone survey of willingness to participate in health research.	Women were more willing to participate than men, and older adults were more willing to participate than young adults. Participants preferred more detailed study explanations to briefer ones.
Halpern et al. (2003)	Motivations for participating or not participating in placebo-controlled trial research study.	Questionnaire using hypothetical research placebo-controlled trials.	Most common motivations for participating included personal health benefits and helping other patients. Most common concerns were having to stop taking current medications and the inconvenience/annoyance of participating.
Kim et al. (2011)	Role of additional information in willingness to consider TMS treatment.	Survey of pregnant women’s perceived acceptability of different mental health treatments (including TMS).	Providing additional information in video and text form increased willingness to consider TMS treatment.
Latimer et al. (2008)	Impact of framing on physical activity participation.	Randomised study using gain, loss, or mixed framing activity guides.	Gain framing resulted in greater physical activity than loss or mixed framing.
Lönnqvist et al. (2007)	Link between personality traits and responses to research study invitation.	Questionnaire using NEO Five-Factor Inventory (NEO-FFI) and Balanced Inventory of Desirable Responding (BIDR).	Study respondents scored lower on neuroticism, and higher on agreeableness and conscientiousness than non-respondents.
Marcus and Schütz (2005)	Personality differences between study respondents and non-respondents.	Combination of self-reported responses to questionnaire and observer ratings using the Big Five Inventory (BFI).	Respondents scored higher on agreeableness and openness to experience than non-respondents.
McDaniel and Zuckerman (2003)	Sensation-seeking and gambling behaviour.	Phone interviews using Impulsive Sensation Seeking Scale (ImpSS) and measures of gambling.	Higher scores on impulsive sensation-seeking linked to greater gambling interest and engaging in more gambling activities.
Oswald et al. (2013)	Personality differences in volunteering for research study participation.	Questionnaires using Revised NEO Personality Inventory (NEO-PI-R), Barratt Impulsiveness Scale (BIS-11), Sensation Seeking Scale (SSS).	Greater sensation-seeking for volunteers compared to non-volunteers.
Otufowora et al. (2021)	Gender differences in willingness to participate.	Questionnaire using seven different research study scenarios.	Men reported greater willingness to participate than women.
Rowland et al. (1986)	Sensation seeking and participation in sports.	Questionnaire using the Sensation Seeking Scale (SSS).	Greater sensation seeking linked to greater participation in sports activity.
Sedlar and Grezo (2022)	Health, demographics, trust, personality, and willingness to participate in biobanking.	Questionnaire using the short Big Five Inventory 2 (BFI-2-S).	Trust in medical researchers linked to greater willingness to participate. No link between personality traits and willingness to participate.
Svensson et al. (2012)	Demographics, attitude, and willingness for parents to enrol children in research studies.	Questionnaire using the Guinea Pig Fear Factor (GPFF) scale and different participation scenarios.	Greatest concerns were fear of their child being “treated as a guinea pig”. Black and Latino respondents reported greater lack of trust in research Parents with lower incomes and less than high school education were most willing to participate in research.
Thomas (1989)	Sensation seeking and willingness to participate.	Questionnaire using Sensation Seeking Scale (SSS) and different study scenarios.	Greater sensation seeking linked to greater willingness to participate when the study was considered “exciting”.
Trauth et al. (2000)	Attitudes and willingness to participate.	Phone survey questionnaire assessing perceptions, attitudes, and willingness to participate.	No effect of gender on willingness to participate.

The present study investigates recruitment challenges for research studies from the perspective of potential participants, specifically within the context of TMS research studies. This was done by assessing perceived barriers and participant concerns, participants’ demographic and personality characteristics, as well as their attitudes towards TMS, willingness to participate, and how these may be related to framing (an introductory text about TMS research studies described in terms of either its theoretical and academic benefits or its potential clinical and treatment benefits).

Based on the literature, we hypothesise that framing the benefits of TMS from a treatment-focused perspective will lead to greater willingness to participate compared to an academic-focused framing perspective. In terms of personal factors, participants who score higher on extraversion, agreeableness, and openness should report a higher willingness to participate. Similarly, those who score higher on sensation-seeking and who report better self-rated health would be more likely to report greater willingness to participate. In terms of barriers, it is hypothesised that potential risks or uncertainty around TMS will be some of the commonly reported concerns and that non-method related factors such as convenience and compensation are also likely to be frequently reported barriers. Finally, it is hypothesised that there will be proportionally more young adults that are eligible based on the safety screening tool than older adults. The data were collected from June 2020 to January 2021, the COVID-19 pandemic, which is likely to be reflected in concerns and barriers to participation.

## Method

### Design

The study comprised an online survey divided into two sections. The first section presented participants with an introductory text describing TMS using one of two framing options for the benefits of TMS. Half of the participants were presented with the first framing option, and the other half with the second framing option. The second section contained the survey questions capturing prior familiarity with TMS, TMS attitudes, medicinal preference, willingness to participate, personality, self-rated health, barriers to participation, and concerns with participating, which were the same for both framing groups.

### Participants

An initial number of 289 participants accessed the survey through volunteer sampling; however, 80 participants were excluded due to incomplete survey submissions, leaving 209 participants (84 male, 124 female, and one non-binary, *M*_age_ = 55.57, SD = 23.93, age range 18–89). Out of these, 105 participants were presented with the academic-focused introduction to TMS (47 males, 58 females, *M*_age_ = 55.28, SD = 24.03) and 104 to the clinical-focused introduction to TMS (37 males, 66 females, one non-binary, *M*_age_ = 55.88, SD = 23.96). Older adults were recruited through existing volunteer panels while young adults were recruited through an existing student recruitment scheme from Nottingham Trent University or via social media advertisement. Participants who were students were compensated via course credits and non-students were offered the opportunity to enter a draw for a £50 Amazon voucher. All participants gave informed consent before commencing the survey.

### Materials

***Framing.*** Both texts began with the same brief description of TMS, how it works, and that it is known to have no detrimental long-term side effects when used safely. The experimental manipulation came in the form of a short text, which described TMS as either a useful method for understanding the brain and building the scientific understanding of brain and behaviour (academic framing), whereas the other text described TMS as a potential treatment for a range of mental health and neurological conditions (clinical framing). The framing text for the TMS introduction that participants read at the beginning of the study was created by the researchers and both versions can be found in Appendix A. Both texts were written in lay terms.

The framing texts were created by the authors and matched as closely as possible in phrasing and word length. The generated texts were provided to colleagues (3) and non-academic (2) individuals for informal feedback, which suggested that the texts were clear and the language appropriate for a general audience.

- ***TMS familiarity, TMS attitudes, and willingness to participate.*** The study asked about participants’ familiarity with TMS using the question: “Did you know what transcranial magnetic stimulation was before this survey?”. The study used three questions to capture attitudes towards TMS: “How invasive do you feel transcranial magnetic stimulation is?”, “How painful do you feel imagine transcranial magnetic stimulation is?”, and “How dangerous do you feel transcranial magnetic stimulation is?” and were rated on a six-point Likert scale from 1 (indicating very painful, very dangerous) to 6 (indicating not at all painful, not at all dangerous). The range of possible scores for this scale was 3 to 18 points. The study assessed willingness to participate using the question “How likely would you be to accept an invitation to participate in a transcranial magnetic stimulation research study?” rated on a seven-point Likert scale with 1 being “Extremely unlikely” and 7 being “Extremely likely”.
- ***Medicinal preference.*** The study included the question “I prefer natural remedies to modern medicine” to assess medicinal treatment preference. The question was rated on a 1–5-point Likert scale with 1 being “Strongly disagree” and 5 being “Strongly agree”.
- ***Barriers to participation and COVID-19.*** The questions relating to barriers of participation had two sections, the first which asked participants to indicate what concerns about TMS made them wish to not participate and the second, what barriers they had to participate. The options were presented as multiple-choice options. These two sections were presented twice, the first one asking them to respond as they would have prior to the COVID-19 outbreak and the second to respond in light of the COVID-19 outbreak, as data was collected during the COVID-19 pandemic. The list of options can be found below in Table 2. If participants chose the “Other” option, they were able to elaborate using an integrated text box which would appear when the “Other” option was selected.

**Table 2:** Table showing the options for concerns about (*left column*) and perceived barriers to (*right column*) participating in TMS research studies

“I would not want to participate in transcranial magnetic stimulation research studies due to concerns about (select as many as you want)”	“ I consider these a barrier to participating in transcranial magnetic stimulation research studies (select as many as you want)”
Having equipment near my head	Transport/travel
Having a magnetic field near my head	Fitting participation into my schedule
Having my brain stimulated	Not enough participation incentives
Possible pain	Not interested in the research
Possible short-term side effects	My mental health
Possible long-term side effects	My physical health
Safety of the equipment	Other
Safety of the procedure	Not applicable
My medical history	
Other	
Not applicable

- ***Personality measures.*** The study used the short version of the Big Five Inventory-2 (BFI-2-S) by Soto and John (2017), which measured the Big Five personality traits (agreeableness, conscientiousness, extraversion, negative emotiveness, and open-mindedness) on a five-point Likert-scale with responses ranging from “Disagree strongly” to “Strongly agree”. The scale used questions such as “I am someone who… Tends to be quiet” and “I am someone who… can be somewhat careless.”. The range of possible scores for each trait was 6–30. The Cronbach’s alpha for this scale reported by its authors to range from 0.73 to 0.83 (Soto & John, 2017).
- ***Self-rated health.*** The study used the Self-rated Health Measure created by Sargent-Cox et al. (2008). This scale included three questions about participants’ health at present (scored from 1 to 5), in comparison to peers (scored from 1 to 3), and in comparison to 12 months ago (scored from 1 to 3). The range of possible scores was 3 to 11, where a lower score indicated better self-rated health and a higher score indicated poorer self-rated health.
- ***Sensation-seeking.*** The study used the impulsivity, risk-tasking, and sensation-seeking scale created by Schafer et al. (1994). The scale contained 11 items and used a four-point Likert scale, where 1 indicated “Not at all” and 4 “Quite a lot”. The scale included questions such as “I often act on the spur-of-the-moment without stopping to think.” and “I like to try new things just for excitement.”. The range of possible scores was 11–44, where a higher score indicated more sensation-seeking behaviours. The Cronbach’s alpha for this scale reported by its authors was 0.87 (Schafer et al., 1994).
- ***TMS Safety Screening Questionnaire.*** The questionnaire used to assess the eligibility for potential TMS research studies was based on the questionnaire from Rossi et al. (2009), covering questions about their medical history and the existence of risk factors, such as epilepsy. Based on their responses, participants were categorised as either “Eligible”, “Not eligible”, or “Uncertain”. Participants who responded “Yes” to whether they were currently taking medication (the types of medicines taken were not reported) or who did not complete all questions were categorised as “Uncertain”.
- ***Survey distribution.*** The survey was created using the Qualtrics software. Participants could access the survey either via a weblink or via the psychology student recruitment system at Nottingham Trent University, SONA, which would redirect them to the survey.

## Procedure

Participants accessed the online survey via a web link. They were presented with an information sheet which included the researcher’s detail in case they wished to ask any questions. After reading the information sheet, participants were presented with the option to provide informed consent and proceed with the study. After this, participants were asked to create a unique identifier in case they wished to remove their data at a later date followed by the survey. The survey presented a brief text explaining transcranial magnetic stimulation, which framed the benefits of TMS research (academic versus clinical) and asked that participants read it carefully before proceeding with the survey. Which text participants were presented with was randomised, showing half the academic and half the clinical framing. Participants were then asked about their familiarity with TMS, questions assessing TMS attitudes, medicinal preference, willingness to participate in a TMS research study, and perceived concerns and barriers. After this, participants completed the Big Five personality inventory followed by the sensation-seeking scale, the self-rated health scale, and the TMS safety screening questionnaire. Finally, participants were asked to report their demographic information after which they were debriefed and thanked for their participation. After the debrief, participants were presented with the option to enter their email address to enter into the voucher draw.

## Results

Based on the safety screening questionnaire, less than a quarter of participants would have been immediately eligible to participate in a TMS research study. Older adults (age 40 or older^1^) were significantly more likely to not be eligible compared to eligible (*X*^2^ (1, *N* = 170) = 26.74, *p* < 0.001) and uncertain compared to eligible (*X*^2^ (1, *N* = 86) = 9.08, *p* = 0.003), as can be seen below in Table 3. A further breakdown of the causes of ineligibility or unknown eligibility can be found in Table 4.

**Table 3:** Eligibility for TMS participation for participants

	Age group		
	Young adults (*N* = 70)	Older adults (40 +) (*N* = 139)	Combined
Eligible	30	17	47
Not eligible	27	96	123
Uncertain	13	26	39

^1^The majority of participants in the older adults group were over 60 with only six aged between 31 and 59. The result excluding middle-aged individuals remained significant when comparing young and older eligible to not eligible (*X*^2^ (1, *N* = 164) = 27.89, *p* < 0.001) and eligible to uncertain (*X*^2^ (1, *N* = 84) = 8.09, *p* = 0.004)

**Table 4:** Breakdown of causes of ineligibility or uncertain eligibility by age group

Safety screening question	*N*	Young adults	Older adults
Have you ever had an adverse reaction to TMS?	0	0	0
Do you have epilepsy or have you ever had a convulsion or a seizure?*	8	6	2
Does any member of your immediate family have epilepsy?	8	3	5
Have you ever had a fainting spell or syncope?*	47	10	37
Have you ever had severe (i.e., followed by loss of consciousness) head trauma?	17	9	8
Do you have any hearing problems or ringing in your ears?**	76	5	71
Are you pregnant or is there any chance that you might be?	0	0	0
Do you have metal in the brain/skull (excluding teeth) or anywhere else in the body? (e.g., splinters, fragments, aneurism clips/coils, etc.)	21	4	17
Do you have cochlear implants?	2	0	2
Do you have an implanted neurostimulator? (e.g., DBS, epidural/ subdural, VNS)	2	0	2
Do you have a cardiac pacemaker or intracardiac lines or metal in your body?	6	1	5
Do you have a medication infusion device?	2	0	2
Are you taking any medications or other drugs/substances?**	114	18	96

These responses are recorded individually per screening question, meaning that a participant may have multiple sources of ineligibility.

* Indicates significance at the *p* < 0.05 level.

** Indicates significance at the *p* < 0.001 level.

The descriptive statistics for the TMS knowledge questions, medicinal preference, and willingness to participate as well as the personality and health scales (Big Five, sensation-seeking, and self-rated health), can be found below in Table 5**.** The results show that most participants were unaware of TMS prior to the survey but held a generally positive attitude. In terms of the reliability of the scales used, Cronbach’s alpha was computed for each of the Big Five traits, sensation-seeking, and TMS attitudes in the sample. The values can be found below. As can be seen, open-mindedness, extraversion, and self-rated health generally had poor reliability. The reliability for TMS attitudes, agreeableness, and conscientiousness was all acceptable, and sensation-seeking showed good reliability.

**Table 5:** Participant responses and mean scores on TMS attitude, personality, and health measures and number of (sub)scale items and Cronbach’s alpha values

Question	Yes	No	Items on (sub)scale	α
Prior TMS familiarity	50	159	-	-
	*M*	*SD*		
Medicinal preference (1–5)	2.45	0.97	-	-
TMS attitude (3–18)	12.98	2.45	3	0.78
Willingness to participate (1–7)	4.91	1.83	-	-
Extraversion (6–30)	19.67	4.61	6	0.46
Agreeableness (6–30)	23.08	4.39	6	0.77
Conscientiousness (6–30)	21.67	4.70	6	0.74
Negative emotiveness (6–30)	15.64	5.54	6	0.84
Open-mindedness (6–30)	22.34	4.05	6	0.44
Sensation-seeking (11–44)	23.79	6.77	11	0.89
Self-rated health total (3–11)	5.93	1.75	3	0.59

### Framing 

Two independent-measures ANOVAs found that there were no effects of framing (academic versus clinical) on willingness to participate (*F*(1, 207) = 4.05, *p* = 0.273, η^2^ = 0.006) or TMS attitudes (*F*(1, 207) = 4.03 *p* = 0.415, η^2^ = 0.003).

### TMS attitude and willingness to participate 

Multiple linear regression was conducted to investigate if medicinal preference, attitudes towards TMS, scores on the Big Five personality traits, sensation-seeking, or self-rated health predicted willingness to participate. The overall regression model was significant (*F*(9, 199) = 13.61, *p* < 0.001, adjusted *R*^*2*^ = 0.35), and inspections of each predictor revealed that more positive attitude towards TMS significantly predicted greater willingness to participate (*p* < 0.001). In terms of the personality factors, conscientiousness was a significant negative predictor of willingness to participate (*p* = 0.022) while no other personality predictors were significant (*p* > 0.05). The VIF scores for the individual predictors indicated a small to moderate correlation between the different measures. The full results can be found below in Table 6

**Table 6:** Results from individual predictors of willingness to participate from the multiple regression

Predictor	Standardised β	Std. error	*t*	*p*	VIF
TMS attitude **	0.60	0.04	10.25	< 0.001	1.10
Medicinal preference	0.07	0.11	1.24	0.217	1.10
Extraversion	0.60	0.03	1.67	0.097	1.44
Agreeableness	– 0.05	0.03	– 0.85	0.399	1.22
Conscientiousness *	– 0.15	0.03	– 2.27	0.022	1.39
Negative emotionality	– 0.10	0.02	– 1.61	0.108	1.25
Open-mindedness	0.09	0.03	1.44	0.151	1.14
Sensation-seeking	– 0.11	0.02	– 1.67	0.097	1.33
Self-rated health	0.07	0.06	1.15	0.250	1.18

* Indicates significance at the *p* < 0.05 level

** Indicates significance at the *p* < 0.001 level

As a relationship between attitude and willingness to participate had been identified, a similar multiple regression was conducted to investigate if scores on the Big Five personality traits, sensation-seeking, or self-rated health predicted TMS attitude. The analysis found no overall significance of the regression model (*F*(7, 201) = 1.36, *p* = 0.226, adjusted *R*^*2*^ = 0.01). No individual predictors were significant (*p* > 0.05). The full results can be found below in Table 7.

**Table 7:** Results from individual predictors of TMS attitude from the multiple regression

Predictor	Standardised β	Std. error	*t*	*p*	VIF
Extraversion	– 0.111	0.04	– 1.35	0.178	1.41
Agreeableness	0.033	0.04	0.44	0.663	1.20
Conscientiousness	0.028	0.04	0.35	0.726	1.37
Negative emotionality	– 0.137	0.03	– 1.80	0.074	1.23
Open-mindedness	0.029	0.05	0.39	0.697	1.14
Sensation-seeking	0.097	0.03	1.24	0.218	1.30
Self-rated health	– 0.106	0.11	– 1.43	0.155	1.17

### Familiarity with TMS

Two independent measures ANOVAs were conducted to investigate if previous familiarity with TMS prior to the survey (familiar vs. unfamiliar) affected attitudes or willingness to participate. The ANOVAs revealed that participants with previous knowledge of TMS reported significantly more positive attitudes than those who were unfamiliar (*F*(1, 207) = 9.27, *p* = 0.003, η^2^ = 0.043), but that familiarity did not affect willingness to participate (*F*(1, 207) = 0.02, *p* = 0.892, η^2^ = 0.000).

### Age and gender 

A further investigation into TMS attitudes and willingness to participate by age group (young vs old) using a one-way independent-measures ANOVA identified that older adults reported significantly greater willingness to participate than young adults (*F*(1, 207) = 4.77, *p* = 0.030, η^2^ = 0.023). In contrast, age did not affect TMS attitudes (*F*(1, 207) = 0.44, *p* = 0.507, η^2^ = 0.002).

To assess what role gender may have on TMS attitudes or willingness to participate, two one-way ANOVAs were conducted. The analyses revealed that participants who identified as men had a significantly more positive attitude towards TMS compared to those who identified as women (*F*(2, 206) = 5.07, *p* = 0.007, η^2^ = 0.047), while gender did not affect willingness to participate (*p* = 0.384). As only one participant identified as non-binary, they were not included in this analysis.

### Concerns and barriers 

Finally, the survey captures the barriers and concerns described by participants pre-COVID-19 and in during COVID-19. The number of concerns and barriers that were identified by participants can be found below in Tables 8 and 9.

**Table 8:** Participant concerns regarding participating

Question	Prior to COVID-19	Since COVID-19
Having equipment near my head	9	14
Having a magnetic field near my head	38	40
Having my brain stimulated	42	40
Possible pain	32	30
Possible short-term side effects	60	57
Possible long-term side effects	76	71
Safety of the equipment	31	33
Safety of the procedure	44	47
My medical history	16	15
Other	11	16
Fear	3	2
Uninterested	2	-
Transport	1	-
Fitting into schedule	1	-
COVID-19 related	-	21

**Table 9:** Perceived barriers to participating

Question	Prior to COVID-19	Since COVID-19
Transport/travel	59	87
Fitting participation into my schedule	51	38
Not enough participation incentives	22	23
Not interested in the research	12	12
My mental health	25	30
My physical health	23	39
Other	11	23
Fear	5	2
Do not understand the method	4	3
Age-/mobility-related concerns	2	1
COVID-19 related	-	14

## Discussion

This project set out to investigate TMS research study participation from the perspective of potential participants to deepen our understanding of the recruitment challenges faced by researchers in the field. The study found that only a minority of participants would have been eligible to participate based on the safety screening questionnaire, should they have been willing to do so, and that this minority was significantly smaller in older adults. TMS has several criteria which could make it unlikely that an older adult would be eligible to participate, nonetheless, it was still remarkable to find so few eligible participants. Notably, the main causes of ineligibility or uncertain eligibility were medications, hearing difficulties, and a history of loss of consciousness, all of which may be more frequently occurring in older adults (Heine et al., 2013; Kenny et al., 2013; Rotermann et al., 2014). This can be challenging for researchers to mitigate, particularly for older adults, as these possible TMS safety concerns can occur as part of healthy aging. Overall, these findings reflect the difficulties with recruitment for experimental studies using clinical or invasive methods (McDonald et al., 2006; Shavers et al., 2001), where the safety screening requirements are greater, and participants are often less familiar with the method.

## Framing

The results demonstrate that framing did not affect how participants perceived TMS or their willingness to participate in a potential TMS research study. Participants were generally positive towards TMS and willing to participate, and more positive attitude towards TMS was associated with greater willingness to participate. This does not align with previous literature suggesting that framing can influence willingness to participate in activities (Latimer et al., 2008). However, it is worth noting that the framing research referred to here investigated physical activity with tangible benefits for the participants themselves. In contrast, the present study’s framing was focused on the benefits of participation for less immediately tangible outcomes (academia and clinical interventions), which may have affected the effect of framing. It is also possible that the previous findings are less applicable to the present study, as physical activity is generally known as well as more accessible for the individual while TMS research studies are much less known, as can be seen in the majority of participants being unfamiliar with TMS prior to this study.

## Attitude and familiarity

The results highlighted a clear link between attitude and participation, showing that a positive attitude towards TMS was linked to a greater willingness to participate. The measure of attitude included questions about perceived risk and tolerability of TMS, and the clear link shown in this study between attitude and willingness to participate echoes findings from the literature (Chu et al., 2015). Despite only few participants being familiar with TMS prior to participating in either study, the results clearly showed that familiarity was linked to more positive TMS attitudes. This is aligned with existing research, which has demonstrated the positive effects of providing additional information on participation (Glass et al., 2015; Kim et al., 2011). Considering previous literature and the present findings, it is likely that a more positive attitude increases willingness to participate, and that this may be mediated by familiarity or understanding reduced perceived risk of TMS. This would align with the findings of Bouida et al. (2016), which demonstrated perceived risk as a key barrier to willingness to participate. However, the present study cannot offer conclusions regarding causal directionality due to its survey design. Additionally, as this project only presented information in text format, it cannot ascertain if the mode of information presentation affects its impact on the willingness to participate. Future research could investigate different modes of presentations (e.g., video, infographic) to understand further what strategies can be employed for more successful study recruitment.

## Personality and demographics

In terms of personality factors, only conscientiousness was found to be a significant negative predictor of willingness to participate. These findings do not align with previous research suggesting a relationship between other personality traits (Ackermann, 2019; Lönnqvist et al., 2007; Marcus & Schütz, 2005) and sensation-seeking (Oswald et al., 2013; Thomas, 1989) and willingness to participate. While Lönnqvist et al. (2007) did find a relationship between conscientiousness and willingness to participate, this was a positive relationship, in direct contrast to the present findings. It is possible that the negative relationship between conscientiousness and willingness to participate in the present study could reflect a general perception of participating in research studies during the COVID-19 pandemic to be a form of non-adherence to the restrictions present at the time of data collection. This would align with literature showing a relationship between conscientiousness and greater adherence to COVID-19 restrictions (Krupic et al., 2021; Turk et al., 2023). Conversely, the findings do largely align with Sedlar and Grezo (2022), suggesting that personality traits may not be as much of a key factor in willingness to participate as previously thought. It is important to note that as data was collected during the COVID-19 pandemic, many participants were experiencing unusually stressful circumstances, which may have influenced responses to certain personality questions commonly associated with certain traits such as openness to new experience, extraversion, and sensation-seeking.

As research is an integral step in developing clinical interventions, it can be reassuring that early stages of TMS research are not subject to notable personality bias in volunteers, aside from the conscientiousness effect reported here, which may be accounted for by the COVID-19 context. Continuing to investigate the role of personality in willingness to participate in TMS studies and receive TMS treatment is valuable for the field, particularly in the light of research suggesting that personality factors may modulate the effects of TMS (Berlim et al., 2013). Future research should continue to investigate the role of personality in willingness to participate in TMS studies as well as receiving TMS treatment, as these may differ and could have potential clinical implications.

The literature on age and willingness to participate did not present a clear picture (Bouida et al., 2016; Glass et al., 2015). This study found that older adults were significantly more willing to participate than young adults. As perceived self-relevance can influence willingness to participate (Glass et al., 2015), and older adults experience (or expect to experience) more health-related ailments that could potentially be treated with TMS, such as dementia (Cantone et al., 2014) and Parkinson’s disease (Nardone et al., 2020). Older adults may perceive participating in TMS research as more self-relevant than young adults.

The present study also investigated how gender is related to willingness to participate. As with aging, the existing literature contains somewhat contradicting evidence (Glass et al., 2015; Green et al., 2011; Otufowora et al., 2021). The findings suggested that participants who identified as men had a significantly more positive attitude toward TMS compared to those who identified as women. While this may initially be a clear indication of men demonstrating a greater willingness to participate, this should be considered in the context of the mixed findings on gender effects on participation (Ding et al., 2007; Glass et al., 2015; Otufowora et al., 2021; Trauth et al., 2000). Based on the previous literature and the present findings, it is likely that a more positive attitude increases willingness to participate. However, the present study is unable to offer conclusions regarding causal directionality due to its survey design.

It is also prudent to consider the context in which the data was collected. Data was collected during the COVID-19 pandemic, meaning that it is possible that these gender differences may be more closely linked to gender differences in COVID-19 risk perception (Alsharawy et al., 2021; Lewis & Dutch, 2021) and adherence to preventative guidelines (Alsharawy et al., 2021; Rana et al., 2021). Finally, this data was collected using online surveys, which may have varying accuracy and generalisability (Singh, 2011), even if questions regarding medical history may be subject to fewer accuracy issues (Kelstrup et al., 2014). The findings should be interpreted with this in mind.

## Practical recommendations

The study’s findings indicate that improving the awareness and perceptions of TMS could boost recruitment. Researchers could invest more resources into public engagement activities, such as talks or live demonstrations. Researchers could also consider providing additional information about TMS or presenting their existing information in a more accessible manner. As some participants also highlighted a lack of interest in the research and the literature suggests that perceived personal relevance can affect willingness to participate (Glass et al., 2015; Moorcraft et al., 2016), researchers could consider how they describe the potential benefits of their research study to potential participants. Another possible avenue researchers could potentially use to increase recruitment is by increasing research participation incentives such as course credits or monetary compensation, which has been recommended by other literature (Treweek et al., 2013). However, the ability of individual researchers to address these challenges and proposed solutions can vary greatly.

What is also important to consider is the potential sampling biases that may occur when recruiting volunteer participants. As outlined in this work, the literature indicates that individuals of certain demographics (Glass et al., 2015) or with certain personalities (Ackermann, 2019) may be more likely to volunteer than others. It is possible that sampling biases may be greater for research studies with greater perceived risks by participants; participants who are more likely to not be as concerned by risk may be overrepresented in these studies. However, it is not clear how this may apply to other forms of research studies. Future research could consider comparing the measures of attitude, concern, barriers, and personality for different methods such as surveys, behavioural studies, neuroimaging studies, TMS studies, and drug intervention studies to further explore the link between attitude, perception, and participation behaviour. Researchers could aim to mitigate potential challenges with sampling by increasing their potential participant pools, for example by creating or expanding existing volunteer participant panels by positively engaging with the local community. However, it is important to note that the considerations around factors leading to participation investigated and discussed here are necessarily drawn from and grounded in the perspectives of researchers (for example, factors that can be controlled by researchers). It is acknowledged here that future research may wish to investigate further the perspectives of participants and patients, perhaps from qualitative perspectives or in a setting where research design is conducted in collaboration with participants, since such studies may emphasise other factors not focussed on here, such as therapeutic implications.

Finally, the proportion of eligible participants was especially low with older adults and researchers seeking to conduct aging research in TMS such as cognitive research or research related to dementia (cf. Cantone et al., 2014; Dong et al., 2018) may need to budget for increased recruitment costs and more stringent background testing to establish suitably sized samples that are representative of the underlying population.

## Electronic supplementary material

Below is the link to the electronic supplementary material.

Supplementary file1 (DOCX 16 KB)

Supplementary file2 (XLSX 30 KB)

Supplementary file3 (PDF 248 KB)

## Data Availability

The datasets analysed during the current study are available in anonymised format in the OSF repository, https://osf.io/sjf7h/.

## Appendix A

### Academic framing

Please read carefully: Transcranial magnetic stimulation is a technique that can stimulate small regions of the brain. A plastic coil unit is placed on the scalp and applies brief magnetic pulses that affect brain activity. Transcranial magnetic stimulation has no known detrimental side effects when it is used within established safety parameters. We can use transcranial magnetic stimulation to actively influence brain activity instead of just observing activity. This helps advance our scientific understanding of how the brain works under different circumstances. We can use it to understand which areas influence which behaviors and how different brain regions work together to perform mental and physical tasks.

### Clinical framing

Please read carefully: Transcranial magnetic stimulation is a technique that can stimulate small regions of the brain. A plastic coil unit is placed on the scalp and applies brief magnetic pulses that affect brain activity. Transcranial magnetic stimulation has no known detrimental side effects when it is used within established safety parameters. Transcranial magnetic stimulation has potential as a treatment for a range of mental health and neurological conditions. It has shown promise as an alternative to medication for treating symptoms of depression, especially in drug-resistant depression. It is also being researched as a potential treatment for symptoms of Parkinson’s disease and dementia, including Alzheimer’s disease.

## The role of individual differences and attitude in willingness to participate in TMS studies: Metadata

Lolansen, C., Howard, C. J., Mitra, S., & Badham, S. P.

This metadata document contains descriptions of the data included in the files ***Study Data***, which was collected between June 2020 and January 2021. For scale measures that do not use pre-existing scales, an additional explanation has been provided.

## Study 1

- **Participant:** Participant number
- **TMSFamiliarity**: Participant was familiar with TMS prior to the study (Yes = 1, 2 = No)
- **TMSReceived:** Participant had received TMS prior to the study (Yes = 1, 2 = No)
- **TMSOtherReceived**: Someone the participant knew had received TMS prior to the study (Yes = 1, 2 = No)
- **MedicinalPref**: Preference for natural remedies to modern medicine measured on a five-point Likert scale with 1 being “Strongly disagree” and 5 being “Strongly agree”
- **TMSAttitude**: Attitude towards TMS measured using three different questions, each rated on a six-point Likert scale. The attitude score was the sum of scores for the three questions with a high score indicating a more positive attitude towards TMS. Scores range from 3 to 18 points
- **WillingnessParticipate**: Willingness to participate in TMS research study measured using a seven-point Likert scale with 1 being “Extremely unlikely” and 7 being “Extremely likely”
- **PreCOVID_Concerns:** Concerns participants indicated having when considering participating prior to the COVID-19 pandemic
- **PreCOVID_Concerns_Other**: Elaboration if participants chose the “Other” option to the previous question
- **PreCOVID_Barriers**: Barriers participants indicated having when considering participating prior to the COVID-19 pandemic
- **PreCOVID_Barriers_Other**: Elaboration if participants chose the “Other” option to the previous question
- **PostCOVID_Concerns**: Concerns participants indicated having when considering participating in light of the COVID-19 pandemic
- **PostCOVID_Concerns_Other:** elaboration if participants chose the “Other” option to the previous question
- **PostCOVID _Barriers**: Barriers participants indicated having when considering participating in light of the COVID-19 pandemic
- **PostCOVID _Barriers_Other:** Elaboration if participants chose the “Other” option to the previous question
- **Extraversion**: Scores on the extraversion component of the Big Five scale
- **Agreeableness**: Scores on the agreeableness component of the Big Five scale
- **Conscientiousness**: Scores on the conscientiousness component of the Big Five scale
- **Negative_Emotiveness:** Scores on the negative emotiveness component of the Big Five scale
- **Open_Mindedness:** Scores on the open-mindedness component of the Big Five scale
- **Sensation_Seeking:** Scores on the Sensation-Seeking Scale
- **SRHealth**: Scores on self-rated health scale
- **TMSEligible**: Eligibility based on TMS safety questionnaire with 1 = Eligible, 2 = Not eligible, and 3 = Unknown. Unknow included participants who had not answered all parts of the safety screening questionnaire or whose only potential contraindication was taking medicine (the exact medicines were not recorded)
- **Age**: Participant age in years
- **Gender**: Participant gender (1 = Male, 2 = Female, 3 = Non-binary)
- **Framing**: Option 1 refers to the academic framing, 2 to the clinical framing
- **AgeGroup:** 0 refers to young adults (under 40) and 1 to older adults (40 and older)

## Declarations

### Open practices statement

The datasets analysed during the current study are available in anonymised format in the OSF repository, https://osf.io/sjf7h/.
