# Supplementary material for: The role of individual differences and attitude in willingness to participate in TMS studies
Source: Behav Res Methods. 2025 Mar 10;57(4):110. doi: 10.3758/s13428-025-02623-4 (PMC11893711; doi:10.3758/s13428-025-02623-4)
Supplement: Supplementary file 1 — Supplementary file1 (DOCX 16 KB) [file 13428_2025_2623_MOESM1_ESM.docx]

The Role of Individual Differences and Attitude in Willingness to Participate in TMS Studies: Meta Data

Lolansen, C., Badham, S. P., Mitra, S., & Howard, C. J.

This meta data document contains descriptions of the data included in the files ***Study Data***, which was collected between June 2020 and January 2021. For scale measures which do not use pre-existing scales, additional explanation has been provided.

**Study 1**

- **Participant:** Participant number
- **TMSFamiliarity**: Participant was familiar with TMS prior to the study (Yes = 1, 2 = No)
- **TMSReceived:** Participant had received TMS prior to the study (Yes = 1, 2 = No)
- **TMSOtherReceived**: Someone the participant knew had received TMS prior to the study (Yes = 1, 2 = No)
- **MedicinalPref**: Preference for natural remedies to modern medicine measured on a 5 point Likert scale with 1 being “Strongly disagree” and 5 being “Strongly agree”.
- **TMSAttitude**: Attitude towards TMS measured using 3 different questions, each rated on a 6-point Likert scale. The attitude score was the sum of scores for the three questions with a high score indicating a more positive attitude towards TMS. Scores range from 3-18 points.
- **WillingnessParticipate**: Willingness to participate in TMS research study measured using a 7-point Likert scale with 1 being “Extremely unlikely” and 7 being “Extremely likely”.
- **PreCOVID_Concerns:** Concerns participants indicated having when considering participating prior to the COVID-19 pandemic
- **PreCOVID_Concerns_Other**: Elaboration if participants chose the “Other” option to the previous question
- **PreCOVID_Barriers**: Barriers participants indicated having when considering participating prior to the COVID-19 pandemic
- **PreCOVID_Barriers_Other**: Elaboration if participants chose the “Other” option to the previous question
- **PostCOVID_Concerns**: Concerns participants indicated having when considering participating in light of the COVID-19 pandemic
- **PostCOVID_Concerns_Other:** elaboration if participants chose the “Other” option to the previous question
- **PostCOVID _Barriers**: Barriers participants indicated having when considering participating in light of the COVID-19 pandemic
- **PostCOVID _Barriers_Other:** Elaboration if participants chose the “Other” option to the previous question
- **Extraversion**: Scores on the extraversion component of the Big Five scale
- **Agreeableness**: Scores on the agreeableness component of the Big Five scale
- **Conscientiousness**: Scores on the conscientiousness component of the Big Five scale
- **Negative_Emotiveness:** Scores on the negative emotiveness component of the Big Five scale
- **Open_Mindedness:** Scores on the open-mindedness component of the Big Five scale
- **Sensation_Seeking:** Scores on sensation seeking scale
- **SRHealth**: Scores on self-rated health scale
- **TMSEligible**: Eligibility based on TMS safety questionnaire with 1 = Eligible, 2 = Not eligible, and 3 = Unknown. Unknow included participants who had not answered all parts of the safety screening questionnaire or whose only potential contraindication was taking medicine (the exact medicines were not recorded).
- **Age**: Participant age in years
- **Gender**: Participant gender (1 = Male, 2 = Female, 3 = Non-binary).
- **Framing**: Option 1 refers to the academic framing, 2 to the clinical framing
- **AgeGroup:** 0 refers to young adults (under 40) and 1 to older adults (40 and older).
