# Supplementary material for: The role of individual differences and attitude in willingness to participate in TMS studies
Source: Behav Res Methods. 2025 Mar 10;57(4):110. doi: 10.3758/s13428-025-02623-4 (PMC11893711; doi:10.3758/s13428-025-02623-4)
Supplement: Supplementary file 3 — Supplementary file3 (PDF 248 KB) [file 13428_2025_2623_MOESM3_ESM.pdf]

Multiple Linear Regression Assumptions Data

This file contains SPSS output analysing the assumptions for the multiple linear regressions performed on the data file in this project.

Willingness to participate

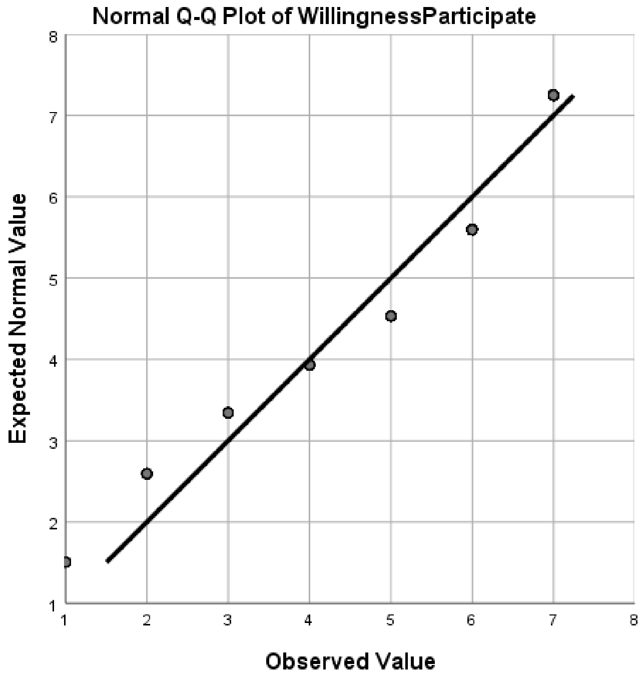

TMS Attitude

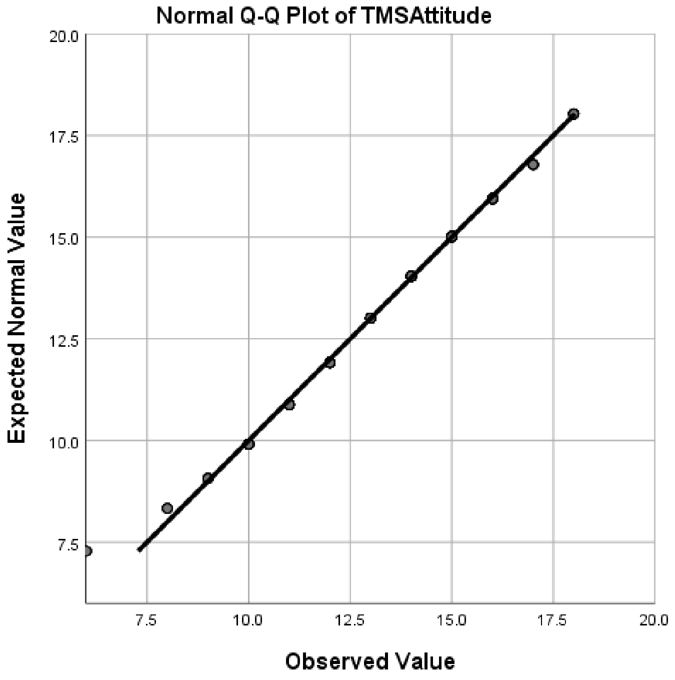

Medicinal preference

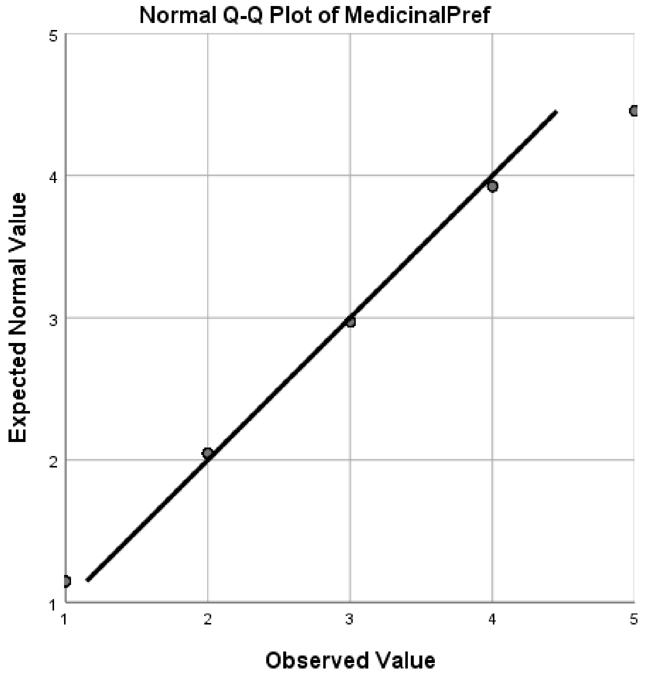

Extraversion

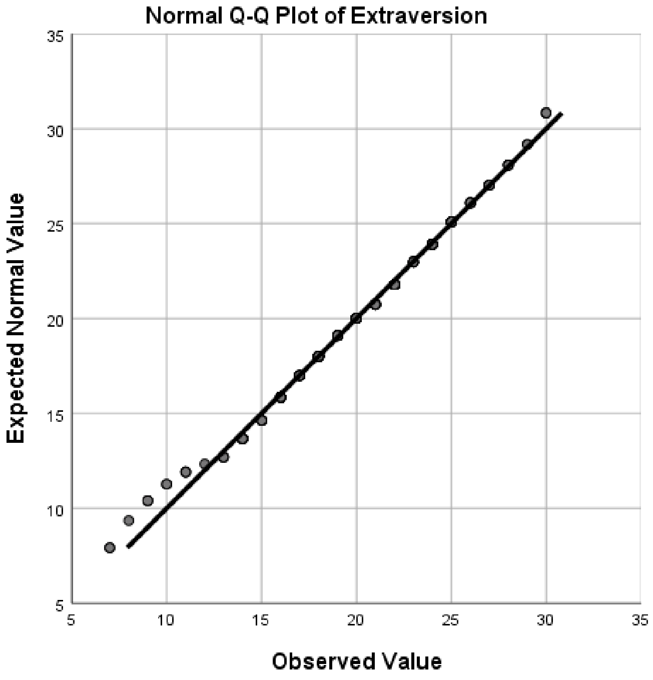

Agreeableness

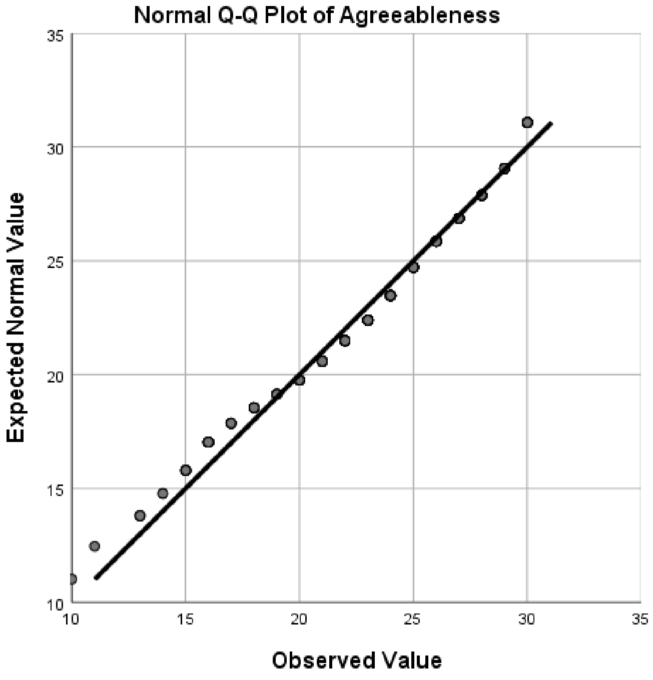

Conscientiousness

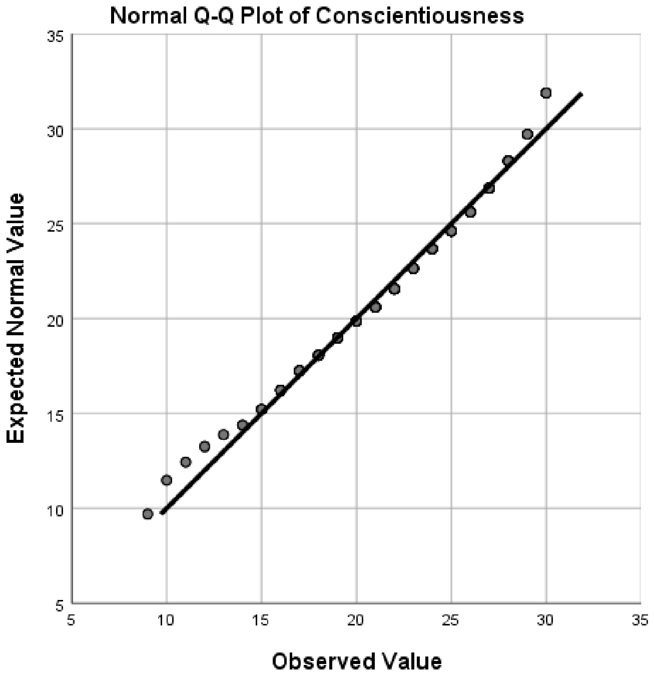

Negative emotiveness

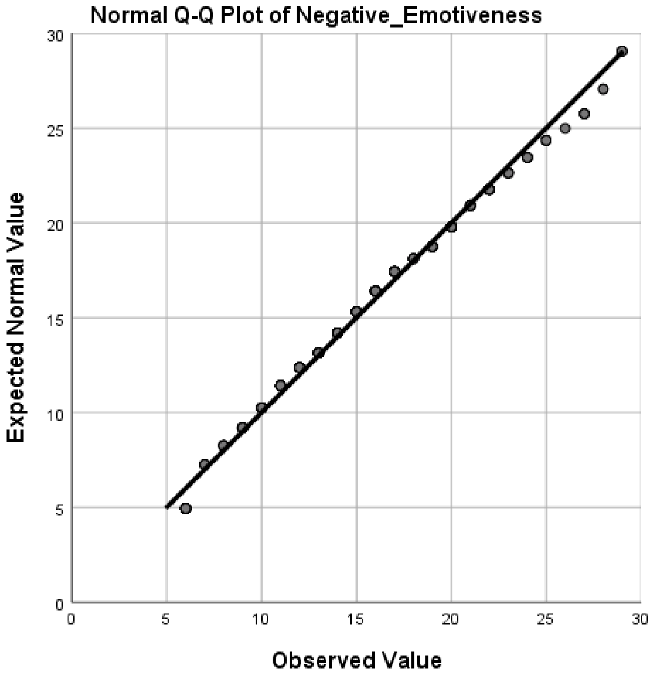

Open-mindedness

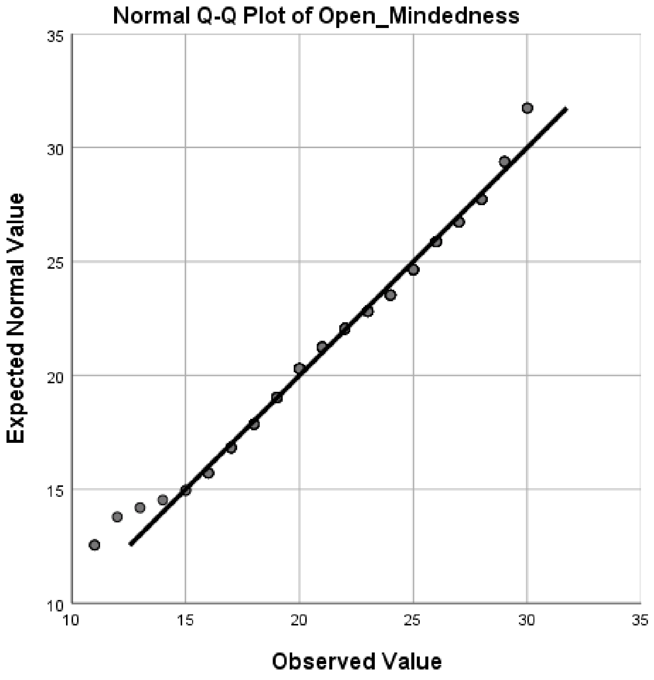

Sensation-seeking

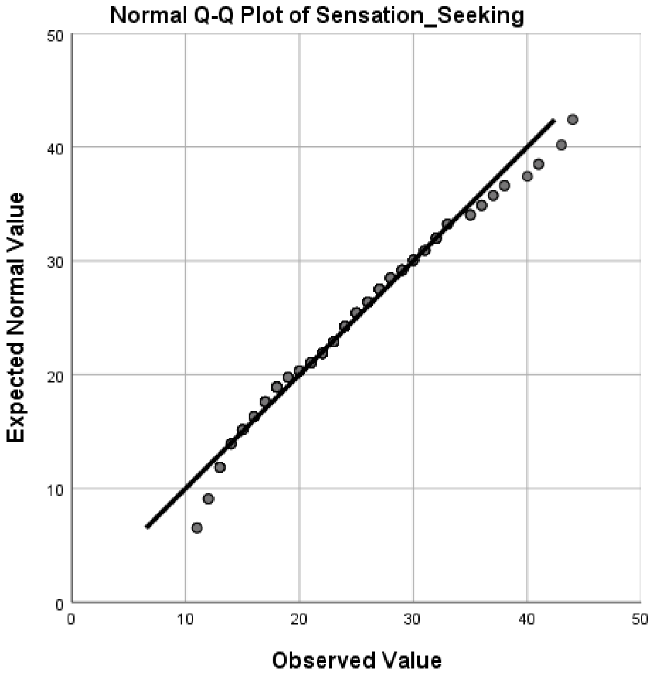

Self-rated health

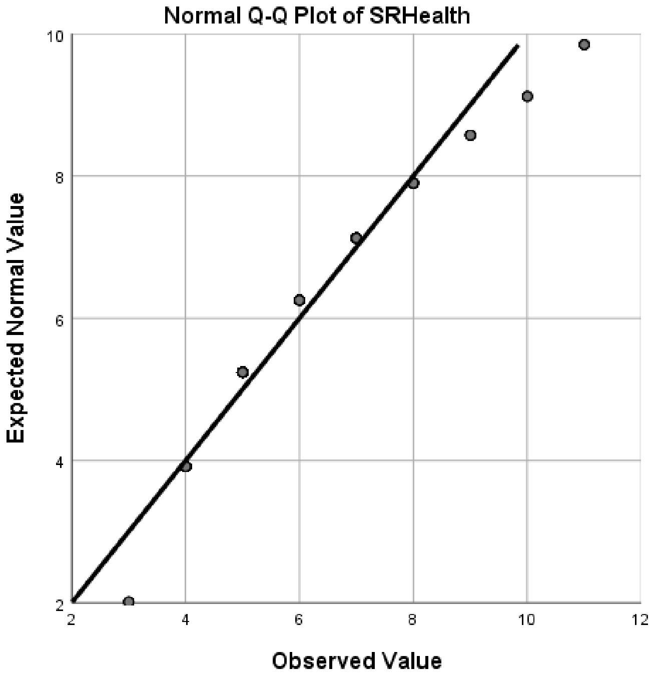

MLR for Willingness to Participate

Coefficients[a]

Model		Unstandardized Coefficients		Standardized Coefficients			Correlations			Collinearity Statistics	
		B	Std. Error	Beta	t	Sig.	Zero-order	Partial	Part	Tolerance	VIF
1	(Constant)	-.154	1.422		-.109	.914					
	Extraversion	.041	.026	.103	1.541	.125	.080	.108	.086	.704	1.420
	Agreeableness	-.018	.026	-.043	-.705	.482	.000	-.050	-.039	.834	1.198
	Conscientiousness	-.054	.026	-.139	-2.125	.035	-.057	-.149	-.119	.730	1.370
	Negative_Emotiveness	-.032	.021	-.098	-1.575	.117	-.164	-.111	-.088	.803	1.245
	Open_Mindedness	.039	.027	.086	1.438	.152	.124	.101	.080	.876	1.141
	Sensation_Seeking	-.027	.017	-.098	-1.535	.126	.037	-.108	-.086	.763	1.311
	SRHealth	.078	.064	.074	1.220	.224	-.014	.086	.068	.848	1.179
	TMSAttitude	.436	.043	.584	10.223	.000	.579	.586	.571	.955	1.047

a. Dependent Variable: WillingnessParticipate

Residuals Statistics[a]

	Minimum	Maximum	Mean	Std. Deviation	N
Predicted Value	1.49	7.53	4.91	1.125	209
Std. Predicted Value	-3.041	2.332	.000	1.000	209

Standard Error of Predicted Value	.139	.554	.298	.073	209
Adjusted Predicted Value	1.51	7.57	4.91	1.128	209
Residual	-5.758	3.178	.000	1.448	209
Std. Residual	-3.899	2.152	.000	.981	209
Stud. Residual	-3.999	2.192	-.001	1.002	209
Deleted Residual	-6.057	3.299	-.002	1.511	209
Stud. Deleted Residual	-4.158	2.214	-.003	1.010	209
Mahal. Distance	.847	28.277	7.962	4.529	209
Cook's Distance	.000	.092	.005	.009	209
Centered Leverage Value	.004	.136	.038	.022	209

a. Dependent Variable: WillingnessParticipate

MLR for TMS Attitude

Coefficients[a]

Model		Unstandardized Coefficients		Standardized Coefficients			Correlations			Collinearity Statistics	
		B	Std. Error	Beta	t	Sig.	Zero-order	Partial	Part	Tolerance	VIF
1	(Constant)	13.995	2.131		6.567	.000					
	Extraversion	-.059	.044	-.111	-1.352	.178	.010	-.095	-.093	.710	1.408
	Agreeableness	.018	.042	.033	.437	.663	.067	.031	.030	.835	1.197
	Conscientiousness	.015	.042	.028	.351	.726	.040	.025	.024	.730	1.369
	Negative_Emotiveness	-.061	.034	-.137	-1.799	.074	-.148	-.126	-.124	.816	1.225

	Open_Mindedness	.017	.045	.029	.390	.697	.052	.027	.027	.877	1.140
	Sensation_Seeking	.035	.029	.097	1.237	.218	.085	.087	.085	.769	1.301
	SRHealth	-.149	.105	-.106	-1.426	.155	-.120	-.100	-.098	.857	1.167

a. Dependent Variable: TMSAttitude

Residuals Statistics[a]

	Minimum	Maximum	Mean	Std. Deviation	N
Predicted Value	11.28	14.56	12.98	.521	209
Std. Predicted Value	-3.264	3.034	.000	1.000	209
Standard Error of Predicted Value	.221	.808	.463	.118	209
Adjusted Predicted Value	11.12	14.92	12.97	.536	209
Residual	-8.161	5.344	.000	2.398	209
Std. Residual	-3.345	2.190	.000	.983	209
Stud. Residual	-3.497	2.234	.000	1.004	209
Deleted Residual	-8.920	5.615	.001	2.501	209
Stud. Deleted Residual	-3.600	2.256	.000	1.010	209
Mahal. Distance	.714	21.800	6.967	4.158	209
Cook's Distance	.000	.142	.005	.012	209
Centered Leverage Value	.003	.105	.033	.020	209

a. Dependent Variable: TMSAttitude
